# Prevalence and treatment of sexually transmitted infections in men followed by urologists in Germany – a cross sectional study with 347,090 men

**DOI:** 10.3205/000265

**Published:** 2018-08-13

**Authors:** Louis Jacob, Dragos Andrei Duse, Karel Kostev

**Affiliations:** 1 Faculty of Medicine, University of Paris 5, Paris, France; 2 University Clinic of Marburg, Germany; 3 Epidemiology, QuintilesIMS, Frankfurt, Germany

**Keywords:** sexually transmitted diseases, prevalence, treatment, urology practices

## Abstract

**Aim:** The goal of this retrospective analysis was to study the prevalence and treatment of the most common sexually transmitted infections (STI) in men followed by urologists in Germany.

**Methods:** This study included a total of 347,090 men followed in 71 urology practices in Germany between 2013 and 2015. The first outcome was the prevalence of individuals diagnosed with STI between 2013 and 2015. The following eight types of STI infections were included in the analysis: chlamydial infection, gonococcal infection, anogenital warts, anogenital herpes infection, trichomoniasis, ulcus molle, phthiriasis, and syphilis. The second outcome was the prevalence of individuals with STI who received an appropriate therapy within 90 days of their initial STI diagnosis.

**Results:** The most frequent STI was anogenital warts (0.64%), whereas the least frequent STI was syphilis (0.03%). The median age at diagnosis ranged from 30.3 years for chlamydia infection to 47.5 years for trichomoniasis. The proportion of individuals receiving treatment was the highest for trichomoniasis (90.6%) and the lowest for anogenital warts (49.9%).

**Conclusions:** Overall, approximately 1.25% of men followed in urology practices in Germany between 2013 and 2015 were diagnosed with at least one STI. Further research is needed to gain a better understanding of the factors potentially associated with the risk of being diagnosed with STI in this setting in Germany. Moreover, there is a need for enabling higher rates of diagnosis and thus treatment of infected persons.

## Zusammenfassung

**Ziel:** Ziel der vorliegenden retrospektiven Studie war es, die Prävalenz und Behandlung der häufigsten sexuell übertragbaren Infektionen (STI) bei in urologischen Praxen in Deutschland beobachteten Männern zu untersuchen.

**Methoden:** Diese Studie umfasste insgesamt 347.090 Männer, die zwischen 2013 und 2015 in 71 urologischen Praxen in Deutschland beobachtet wurden. Das erste Outcome war die Prävalenz von Personen, bei denen zwischen 2013 und 2015 eine STI diagnostiziert wurde. Die folgenden acht Arten von STI wurden in die Analyse aufgenommen: Chlamydieninfektionen, Gonokokkeninfektionen, anogenitale Warzen, Infektionen des Anogenitalbereiches durch Herpesviren, Trichomoniasis, Ulcus molle, Phthiriasis und Syphilis. Das zweite Outcome war die Prävalenz von Personen mit STI, die innerhalb von 90 Tagen nach der ersten STI-Diagnose eine angemessene Behandlung erhielten.

**Ergebnisse:** Anogenitalwarzen traten am häufigsten auf (0,64%), während Syphilis am seltensten diagnostiziert wurde (0,03%). Das durschnittliche Alter zum Diagnosezeitpunkt reichte von 30,3 Jahren (Chlamydieninfektion) bis 47,5 Jahre (Trichomoniasis). Der Anteil der Personen, die eine Behandlung erhielten, war für Trichomoniasis am höchsten (90,6%) und für Anogenitalwarzen (49,9%) am niedrigsten.

**Schlussfolgerungen:** Insgesamt wurde bei circa 1,25% der zwischen 2013 und 2015 in urologischen Praxen in Deutschland beobachteten Männer mindestens eine STI diagnostiziert. Weitere Studien sind erforderlich, um die Faktoren, die möglicherweise mit dem Risiko einer STI-Diagnose in dieser Umgebung in Deutschland assoziiert sind, besser zu verstehen.

## Introduction

More than one million sexually transmitted infections (STI) are acquired in the world each day [1]. Chlamydia, gonorrhea, syphilis, and trichomoniasis are responsible for 25% of the total number of STI diagnosed each year worldwide. With 394,163 new registered cases in 2015, chlamydia infection is the most common STI in EU/EEA Member States [2].

The prevalence of STI in men in Germany has been the focus of only a few studies in the past decade. Although a 2010 study including 14 million insured individuals showed that the estimated incidence rate of anogenital warts was 169.5 cases per 100,000 person-years [3], no recent data on the prevalence of this STI in this country is available. In the case of chlamydia and gonorrhea infections, Dudareva-Vizule and colleagues estimated that the prevalence of these two STI in men having sex with men ranged from 1.5% to 8.0% depending on the anatomical site where samples were collected [4]. These findings were later corroborated by a study conducted in North Rhine-Westphalia in which chlamydia infection was found in 3.2% of heterosexual men and 3.5% of homosexual men [5]. Regarding trichomoniasis, no recent works have analyzed the prevalence of this STI in Germany. Nonetheless, a previous study conducted in the U.S. discovered that trichomoniasis was diagnosed in 1.7% of young men [6]. Finally, studies aimed at estimating the prevalence of syphilis are also lacking. That being said, Bremer et al. found that the incidence of syphilis had significantly increased between 2010 and 2011, as well as that around 3,700 patients were diagnosed with this STI in 2011 [7]. Interestingly, the increase was higher in men (23%) than in women (13%).

Even if the previous results are of great importance, little is known about the prevalence of these STI in men in Germany in recent years. Furthermore, almost no data is available on the share of patients receiving appropriate treatment. Therefore, the goal of this retrospective analysis was to study the prevalence and treatment of the most frequent STI in men followed by urologists in Germany.

## Methods

### Database

Data from the nationwide Disease Analyzer database (QuintilesIMS) were used for the present study. Demographic, clinical, and pharmaceutical data included in this database were obtained from a nationwide sample of general and specialist practices [8]. The quality of the information is regularly assessed by QuintilesIMS, and the representativeness of the Disease Analyzer database for German primary care practices has already been underlined by previous works [8]. The sampling method for the Disease Analyzer database is based on summary statistics from all physicians in Germany published every year by the German Medical Association. The IQVIA uses these statistics to determine the panel design according to specialist group, German federal state, community size category, and age of physician. There are different practices in the panel in terms of patient number (big and small practices) and town size (urban and country).

Finally, several studies focusing on infectious diseases and the prescription of associated treatments have been conducted using this database in the past [9], [10], [11]. 

### Study population and outcomes

This study included men followed in 71 urology practices in Germany between 2013 and 2015. The first outcome was the prevalence of individuals diagnosed with STI between 2013 and 2015. Eight different types of STI infections were included: chlamydial infection (International Classification of Diseases, 10^th^ revision [ICD-10]: A55, A56, A70, A74), gonococcal infection (A54), anogenital warts (A63.0), anogenital herpes infection (A60), trichomoniasis (A59), ulcus molle (A57), phthiriasis (B85.3), and syphilis (A50–53). Since candida infections are often favored by the prescription of antibiotics, diabetes, and disorders associated with immune system deficiencies [12], we did not include these infections in our study. The second outcome was the prevalence of individuals with STI who received an appropriate therapy within 90 days of their initial STI diagnosis. We included four types of treatment: systemic antibiotics (Anatomical Therapeutic Chemical [ATC]: J01), antivirals for dermatological use (D06BB), systemic antivirals (J05), and antiprotozoals (P01).

### Statistical analyses

This study is a cross-sectional retrospective study based on descriptive statistics. The prevalence of STI infections, defined as the proportion of patients diagnosed with STI, was analyzed. We further studied the prevalence of patients receiving therapy in the different STI groups. All analyses were carried out using SAS 9.3 (SAS Institute, Cary, NC, USA).

## Results

**Figure 1:**
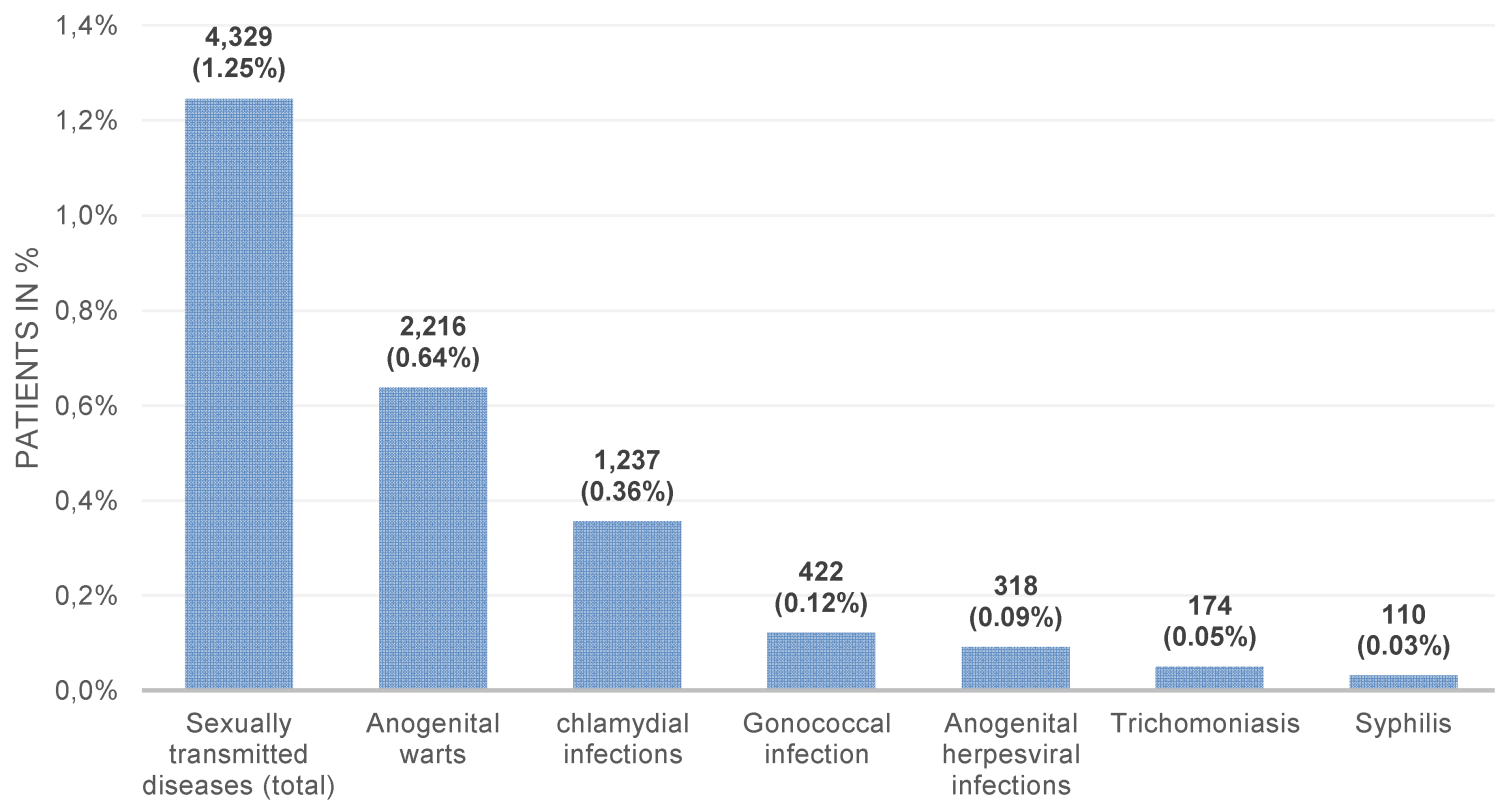
Prevalence of sexually transmitted diseases diagnosed in 347,090 outpatients followed by urologists in Germany between 2013 and 2015

The present study included 347,090 men. Table 1 (Tab. 1) and Figure 1 (Fig. 1) show the prevalence of the six different STI in men followed in urology practices in Germany. Overall, 4,329 patients (1.25% of the population) were diagnosed with at least one STI between 2013 and 2015. The most frequent STI was anogenital warts (n=2,216, 0.64%), whereas the least frequent STI was syphilis (n=110, 0.03%). No cases of phthiriasis and ulcus molle were found.

**Figure 2:**
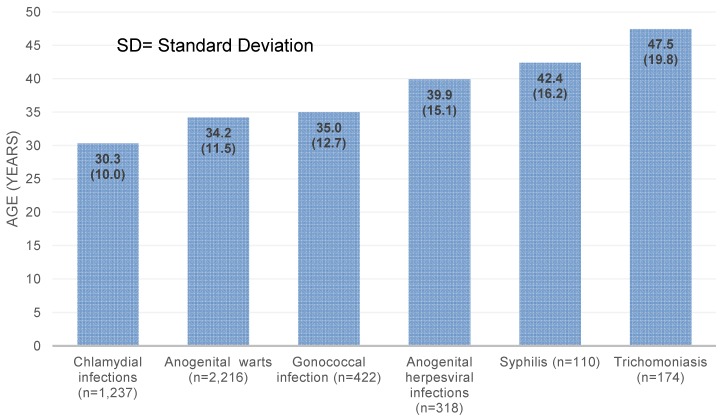
Mean age (and SD) at diagnosis by sexually transmitted disease in men followed by urologists in Germany between 2013 and 2015

The median age at diagnosis ranged from 30.3 years for chlamydia infection to 47.5 years for trichomoniasis (Figure 2 (Fig. 2)). The prevalence of men receiving defined therapy is shown in Table 2 (Tab. 2). The proportion of individuals receiving treatment was the highest for trichomoniasis (90.6%) and the lowest for anogenital warts (49.9%).

## Discussion

In this retrospective study of 347,090 men followed in urology practices in Germany, 1.25% of the population was affected by at least one STI. The most frequent STI was anogenital warts and the least frequent one was syphilis. The mean age at diagnosis ranged from 30 years to 48 years. Finally, the proportion of individuals receiving treatment was the highest for trichomoniasis and the lowest for anogenital warts. 

The most important result of this work is that STI were relatively rare in men followed by urologists in Germany. Before continuing, one has to bear in mind that several of the infections associated with these sexually transmitted microorganisms (i.e. *Chlamydia trachomatis*, *Neisseria gonorrhea*, or *Treponema pallidum*) are often asymptomatic [13], [14], [15], [16]. Therefore, the big part of STI infections may have been undiagnosed. That being said, we found that anogenital warts were the most frequent STI in men followed in urology practices in Germany. A recent review conducted by Patel et al. showed that the prevalence of this disease was lower than 5% throughout the world and varied with the type of database used for the statistical analysis [17]. Interestingly, the prevalence was between 0.2% and 5.1% when genital examinations were available, but only between 0.13% and 0.56% when only administrative or medical databases or prospectively collected physician reports were available. 

Chlamydia infection was the second most common STI in this setting. This finding is in line with previous studies conducted in Germany. A 2011 study including 1,815 urine samples of adolescents estimated that chlamydia was found in less than 1% of the population [18]. The authors further showed that boys were less likely to be diagnosed with this STI than girls (0.1% versus 1.8%). Later, in 2016, Lallemand and colleagues investigated the prevalence of *Chlamydia trachomatis* infection in women, heterosexual men, and homosexuel men visiting HIV counseling institutions in North Rhine-Westphalia [5]. The authors discovered that chlamydia infection was present in 5.3% of women, 3.2% of heterosexual men, and 3.5% of homosexual men. Heterosexual men aged 18–24 years (5.7%) and homosexual men aged 30–39 years (4.4%) were further found to be at a particular risk of being diagnosed with this STI. Compared to the work of Lallemand et al. [5], the prevalence of chlamydia infection was much lower in the present retrospective study. The major difference between these two works is that Lallemand and colleagues focused on people visiting HIV counseling institutions, whereas we were interested in all men followed by urologists in Germany [19]. Therefore, sexual risk behaviors likely differed between these two populations. 

Moreover, we found that 0.12% of men were diagnosed with gonococcal infection. This result is of particular importance because, since most recent studies conducted in Germany have focused on homosexual men, data on heterosexual men is lacking. As the majority of men are heterosexual, we assume that the prevalence analyses performed in this study rather show the prevalence in heterosexual men. In 2014, Dudareva-Vizule et al. estimated in 2,247 homosexual men that the prevalence of *Neisseria gonorrheae* was 5.5% in pharyngeal specimens and 4.6% in rectal specimens [4]. Chlamydia infection was further diagnosed in 1.5% and 8.0% of pharyngeal and rectal specimens, respectively. 

Anogenital herpesviral infection was present in almost 0.1% of men followed by urologists in Germany. Again, the real prevalence of this STI may likely be much higher than we estimated. For example, a 2001 study including 374 patients from Spain discovered that herpes simplex virus type 2 was found in around one out of four patients (12% of men and 30% of women) [20]. This finding was later corroborated in a European work conducted by Pebody and colleagues [21]. This analysis showed that the age-standardized prevalence of this virus was between 4% and 24% in Europe and approximately 14% in Germany. 

The two least frequently diagnosed STI were trichomoniasis and syphilis. In 2000, Joyner and colleagues estimated in 454 men attending a STI clinic that *Trichomonas vaginalis* was detected in 2.8% of the population [22]. They further found that this STI mainly affected individuals aged 30 years and over (5.1%). Finally, the authors discovered that the presence of discharge and nongonococcal urethritis in men ≥30 years increased the risk of being diagnosed with *Trichomonas vaginalis*. In the case of syphilis, no recent data on the prevalence of this STI in Germany is available. Nonetheless, in a recent study Bremer and colleagues found that syphilis incidence increased between 2010 and 2011 [7]. Of particular importance was the fact that the vast majority of new cases were found in men (95%). 

Finally, it is important to note that the prevalence of patients receiving treatments differed between the STI. Interestingly, less than 50% of men with anogenital warts were treated. There are currently multiple options for treating anogenital warts (i.e. topical molecules, cryotherapy, electrosurgery, or CO_2_ laser) [23], [24]. Based on this finding, it seems likely that men with anogenital warts who did not receive drugs were prescribed physical treatments. Apart from the men diagnosed with anogenital warts, the majority of the population received an appropriate treatment, and one can hypothesize that those without treatments were in fact treated directly by general practitioners or surgeons.

This retrospective study was subject to several limitations that should be mentioned at this point. No biological information was available, and each STI diagnosis relied only on ICD-10 codes. The quality of ICD Code documentation and coding itself can vary from physician to physician. Moreover, several STI tests are often not performed due to cost reasons or due to lacking of symptoms. Furthermore, since accessing data obtained in general practices was not possible, the diagnoses were only based on data entered by urologists. Not only general practitioners, but also special centres can play role in the support of STI patients. The reasons for prescriptions were not always documented and it is possible that a part of the prescriptions were given for other infections and not for STI. Finally, no information regarding factors associated with sexual risk behavior (i.e. alcohol, tobacco, and drug use or socioeconomic status) was available. The major strengths of this study are the number of urology practices and the number of patients available for analysis.

Overall, approximately 1.25% of men followed in urology practices in Germany between 2013 and 2015 were diagnosed with at least one STI. Furthermore, we found that between 50% and 83% of the men diagnosed with STI received an appropriate treatment. Further research is needed to gain a better understanding of the factors potentially associated with the risk of being diagnosed with STI in this setting in Germany. Moreover, there is a need for enabling higher rates of diagnosis and thus treatment of infected persons.

## Data

Data for this article are available from the Dryad Repository: https://doi.org/10.5061/dryad.t22jv [25] 

## Notes

### Competing interests

The authors declare that they have no competing interests.

### Ethical approval

All procedures performed in studies involving human participants were in accordance with the ethical standards of the institutional and/or national research committee and with the 1964 Helsinki declaration and its later amendments or comparable ethical standards.

German law allows the use of anonymous electronic medical records for research purposes under certain conditions. According to this legislation, it is not necessary to obtain informed consent from patients or approval from a medical ethics committee for this type of observational study that contains no directly identifiable data. Therefore, no waiver of ethical approval was obtained from an Institutional Review Board (IRB) or ethics committee. The authors had no access to any identifying information at any moment during the analysis of the data. 

### Acknowledgments

Professional English language editing services were provided by Claudia Jones, MA, Radford, Virginia, United States.

## Figures and Tables

**Table 1:**
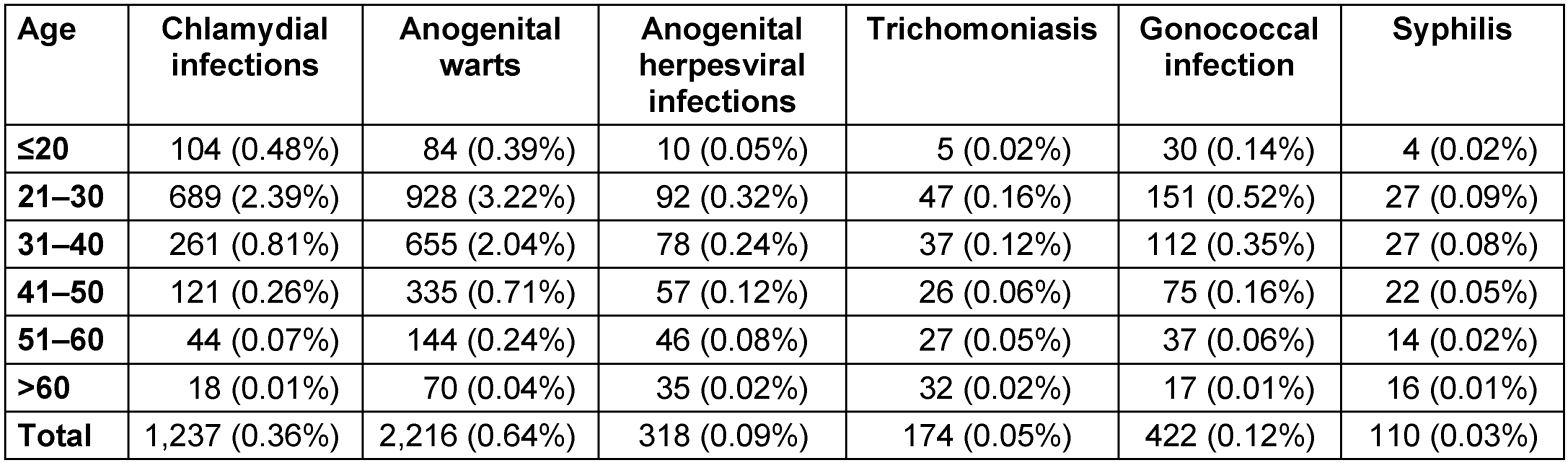
Prevalence of sexually transmitted diseases diagnosed in men followed in urology practices in Germany between 2013 and 2015

**Table 2:**
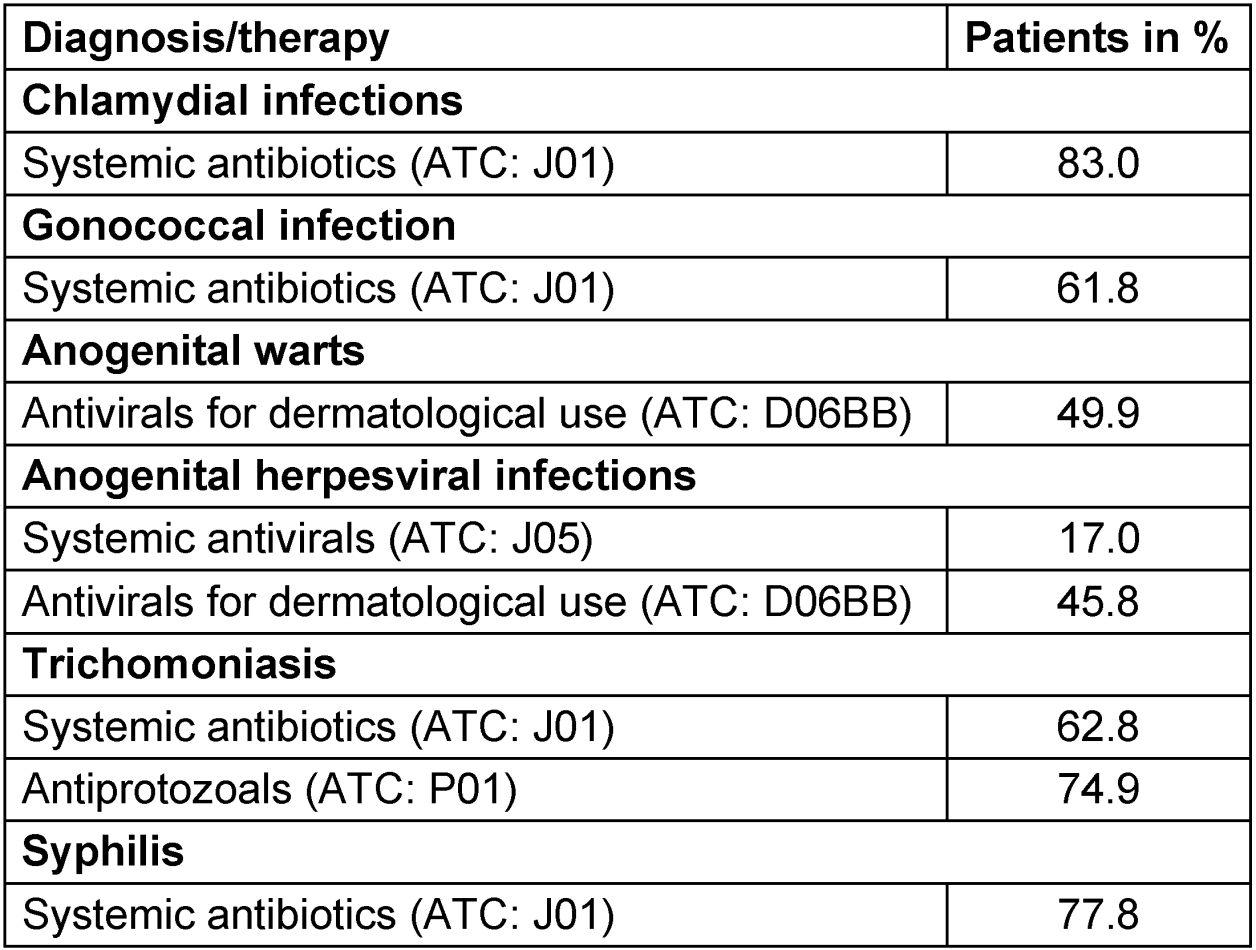
Prevalence of men diagnosed with sexually transmitted diseases and receiving therapy in urology practices in Germany (at least one prescription issued within 90 days after diagnosis was first documented)
